# Cognitive ergonomics and robotic surgery

**DOI:** 10.1007/s11701-024-01852-7

**Published:** 2024-03-05

**Authors:** Shing Wai Wong, Philip Crowe

**Affiliations:** 1 Department of General Surgery, Prince of Wales Hospital, Sydney, NSW Australia; 2 School of Clinical Medicine, The University of New South Wales, Randwick Campus, Sydney, NSW Australia

**Keywords:** Cognition, Ergonomics, Robotic surgery, Cognitive workload

## Abstract

Cognitive ergonomics refer to mental resources and is associated with memory, sensory motor response, and perception. Cognitive workload (CWL) involves use of working memory (mental strain and effort) to complete a task. The three types of cognitive loads have been divided into intrinsic (dependent on complexity and expertise), extraneous (the presentation of tasks) and germane (the learning process) components. The effect of robotic surgery on CWL is complex because the postural, visualisation, and manipulation ergonomic benefits for the surgeon may be offset by the disadvantages associated with team separation and reduced situation awareness. Physical fatigue and workflow disruptions have a negative impact on CWL. Intraoperative CWL can be measured subjectively post hoc with the use of self-reported instruments or objectively with real-time physiological response metrics. Cognitive training can play a crucial role in the process of skill acquisition during the three stages of motor learning: from cognitive to integrative and then to autonomous. Mentorship, technical practice and watching videos are the most common traditional cognitive training methods in surgery. Cognitive training can also occur with computer-based cognitive simulation, mental rehearsal, and cognitive task analysis. Assessment of cognitive skills may offer a more effective way to differentiate robotic expertise level than automated performance (tool-based) metrics.

## Introduction

Ergonomics has been classified as having physical, organisational, and cognitive components. Physical ergonomics relates to the physical body, organisational ergonomics with systems, and cognitive ergonomics with the human brain. Physical ergonomics relate to musculoskeletal discomfort and is usually assessed objectively by electromyography [1]. Organisational ergonomics can be assessed by workflow disruptions [2]. Cognitive ergonomics refer to mental resources and is associated with memory, sensory motor response, and perception [3]. Cognitive workload (CWL) involves use of working memory (mental strain and effort) to complete a task [4]. It is the information processed by the working memory, which is limited in its capacity to process information. Cognitive load theory assumes a limited working memory (7 +/− 2 concurrent information elements) and an unlimited long-term working memory holding cognitive schemas [5]. Working memory are applied to novel information and can process only a small number of elements simultaneously for a few seconds with usually no retention after 20 s unless it is refreshed by rehearsal. The three types of cognitive loads have been divided into intrinsic (dependent on complexity and expertise), extraneous (the presentation of tasks) and germane (the learning process) components.

Cognitive overload can occur because of the high demands of a surgical task as well as factors such as workflow disruptions [6]. This can impair performances and impair decision making especially when performing tasks which are complex or non-routine. Continual practices can lead to automaticity which can allow surgeons to dedicate working memory for more demanding work. The Yerkes-Dodson law (with Hebb’s simplification) suggests that there is an optimal level of CWL corresponding to an optimal level of performance, with an inverted U-shaped relationship between CWL and performance [7]. Task performance is best when mental arousal levels are in the middle range, with difficult tasks best performed under lower levels of arousal and simple tasks best performed under higher levels of arousal. As expertise increase, surgeons can anticipate, pre-empt, and process technical challenges during unexpected operative scenarios [8]. One study reported an association between increase cognitive workload and less errors by experienced surgeons. This may be related to more focus given to a more complex task and developed automaticity during less complex more routine tasks. [9]

The impact of robotic assistance on CWL is complex. On one hand, the better associated postural, visualisation and manipulation ergonomics may facilitate less need to delegate cognitive resources to the physical tasks [10]. On the other hand, CWL may be increased for the robotic surgeon because of reduced situational awareness related to physical separation from the patient and team members, communication difficulties, need to control more instruments, limited visual field and lack of haptic feedback [2]. Intraoperative CWL can be measured subjectively post hoc with the use of self-reported instruments but have the disadvantage of no real-time assessment [11]. Physiological responses that occur with CWL are objective measurements which can provide real-time metrics. They have included measurements of heart rate, skin conductance, electoencephalography, functional near-infrared spectroscopy, and pupil studies.

This review will present the impact of robotic assistance on CWL, as well as the impact of physical and organisational ergonomics on cognition. The range of CWL measurement tools (with their reliability and validity), the benefits of cognitive training, and assessment of cognitive skills will be presented.

## Impact of robotic assistance on CWL

Most recent workload research on robotic surgery focused on physical effort as measured by electromyography. Research into mental effort has been relatively sparse and usually involved simulation studies but is gaining traction. Mental overload especially when multi-tasking is involved, can lead to poor task performance. Studies reporting on the impact of robotic assistance on cognitive workload have not been consistent in their findings. A systematic review reported significantly lower cognitive load in robotic than laparoscopic surgery in seven of ten observational studies [10]. The impact was dependent on factors such as simulation or real setting, surgeon experience, previous laparoscopic experience, robotic technical skills, and assessment used.

Simulation or real setting studies may have different conclusions on CWL, partly because most simulation studies are performed on novice or less experienced surgeons. A simulation robotic surgery study involving novice surgeons found that visual-perceptual mismatch (VPM) impaired performance and added to the cognitive load [12]. VPM is a brain sensory misalignment of the location of the surgeon hands at the robotic console and the location of the robotic instruments as visualised through the console binoculars. In contrast, another study revealed that VPM was well compensated for an experienced robotic colorectal surgeon during real surgery [13]. Despite the additional cognitive load of VPM, ergonomic side-by-side hand positioning was preferred. The experienced surgeon may be able to combine different unimodal sensory cues from a common source to a single multisensory estimate which minimises variance [14].

Lower subjective mental workload in novice and experienced surgeons when operating with robotic assistance have been reported in most but not all simulation studies [15–17]. Moore et al reported surgeons experiencing less total workload when performing tasks on the robotic system compared to the laparoscopic system [15]. The authors attributed this less mental effort investment to less stressful, physically demanding, and complex surgery on the robotic system. The authors postulated that surgeon working memory may be less stretched by using robotic technology which can leave more cognitive resources to help them deal with other demands such as decision making. Huxhold et al. postulated that the reduced need to divert cognitive resources for postural control during robotic surgery may allow surgeons to focus on the surgical task [18]. In contrast, Lau et al. reported no improvement in subjective mental workload with robotic assistance for novice surgeons, which they attributed to inadequate pre-study robotic training [19]. In most studies of experienced surgeons in real and simulated settings, robotic assistance has been shown to reduce mental workload when compared with laparoscopic surgery [20, 21].

Surgeons experienced in both laparoscopic and robotic surgery reported lower cognitive workload with robotic assistance whereas the opposite was true for surgeons with only laparoscopic experience and no robotic experience [3, 22, 23]. The higher workloads with robotic surgery experienced by pure laparoscopic surgeons was attributed to their familiarity and expertise with laparoscopic surgery only [3]. In contrast, Shugaba et al. reported greater cognitive demands during robotic surgery compared with laparoscopic surgery. In this study, different surgeons performed, depending on their expertise, either laparoscopic or robotic real-life surgery but not both. The increased cognitive demand with robotic surgery was attributed to greater demand on visual attention systems partly related to loss of haptic feedback and increased mental fatigue as evidenced by alpha and beta desynchronization on EEG monitoring [24].

Roberts et al. reported different associations between technical skills and CWL based on surgeon experience levels [9]. They found that there was an optimal CWL level for surgeons of all experience levels. As more difficult technical skills as assessed by the validated Global Evaluative Assessment of Robotic Skills was performed, CWL decreased in novice surgeons but increased in experienced surgeons. More errors were committed by novice surgeons with increasing CWL but less errors were committed by experienced surgeons with increasing CWL. The authors attributed their findings to experienced surgeons working at a lower CWL at baseline because of developed automaticity.

The subjective or objective cognitive assessment used can have an impact on the results. Hubert et al observed no difference in cognitive fatigue using NASA-TLX scores between laparoscopic and robotic surgery but noted lower fatigue scores with robotic surgery when assessment was performed objectively with physiological heart rate metrics [25]. To explain their findings, the authors postulated that surgeons were potentially more sensitive to the physical than the mental stress when they were questioned after completion of surgery.

## Impact of fatigue on CWL

Longer operative times contribute to surgeon mental and physical fatigue, which can lead to reduction in fine motor control and precision [26]. Human performance has been shown to be impaired by fatigue from prolonged work hours. Fatigue typically sets in when surgical time approaches four hours with symptoms including mental exhaustion, irritability and impaired surgical judgement [27]. One study involving 15 surgeons performing exercises on the robotic simulator found that after 2 h, a statistically significant deterioration in performance was observed and this continued to worsen until exercise termination 4 h later [28]. A meta-analysis and other studies have revealed cognitive performance to be more impaired by fatigue than psychomotor skills [29, 30]. The studies concluded that there should be a greater emphasis in preventing cognitive errors during times of fatigue. Cognitive fatigue has been shown to be associated with impaired performance related to slower reaction times, reduction in concentration and impaired memory and information processing [10].

## Impact of workflow disruptions on CWL

The separation of the surgeon from the bedside team during robotic surgery has been demonstrated to have a negative effect on the CWL of the team members. Interpersonal cues, verbal and non-verbal communication, and team dynamics are impaired or altered. Team members need to adjust communication, teamwork, and co-ordination skills with separation of the surgeon. Reduced surgeon situation awareness can be compensated for by other team members with shared mental models which requires trust, effective communication, customs, and practice [31]. Better anticipation by the team during robotic surgery was shown to be associated with lower surgeon cognitive load, as measured by NASA Task Load Index. [32] Completion of anticipated requests were five times faster than non-anticipated requests (5 and 26 s, respectively).

Zamudio et al performed a prospective study quantifying the workload experienced by all surgical team member during robotic surgery [33]. They found that surgeons and circulating nurses reported the highest workload. They attributed this to the extensive human–robot interactions expected of surgeons and the many responsibilities of the circulating nurse outside the sterile fields which included coordinating tasks within and outside the operating room and ensuring all necessary equipment was readily available. The role in the surgical team affects the perceived stress and workload during surgery. Cavuoto et al. found that physical demands and temporal demands were highest for the bedside assistant, and this was related to their role in assisting with surgeon requests in a timely manner and to prevent interruptions [34]. They found that the scrub nurses had the lowest workload scores, which were attributed to their experience as well as their less stressful role in ensuring availability of instruments during robotic surgery, which they can share with the bedside assistant.

## Measurement tools

Workload represents the psychological cost to perform a task and is human-specific. Workload measurements can be performed by subjective self-rating techniques, objective non-technical skill assessment metrics, or objective physiological signals [35]. The two most used mental workload assessment techniques are the NASA Task Load Index (NASA-TLX) and the related Surgery Task Load Index (SURG-TLX). The NASA-TLX is a validated multidimensional self-perceived assessment tool relating to the domains of mental demand, physical demand, temporal demand, performance, effort and frustration [36]. The SURG-TLX measure also assesses six workload dimensions involving mental demand, physical demand, temporal demand, task complexity, situational stress, and distractions [37]. Self-report techniques may be less reliable because it is usually administered after the procedure and CWL may vary in intensity and duration during different phases of the surgery.

Other available mental workload assessment techniques have been used to assess multitasking skill, mental effort, physical discomfort, communication, job satisfaction, and cognitive strategy adaptation. Expert rating techniques can assess non-technical skill more objectively but is still subject to bias. The Interpersonal and Cognitive Assessment for Robotic Surgery (ICARS) was developed to specifically measure the non-technical skills of robotic surgeons [38]. ICARS measures three main non-technical skill categories: interpersonal skills (communication, teamwork, and leadership), cognitive skills (decision making and situational awareness) and personal resources skills (coping with stress and distractions). ICARS measures several unique behaviours which are not described in other generic rating systems including awareness of team members, patient status, and equipment failure whilst at the surgeon console.

Real-time assessment methods are classified as physiological metrics and include heart rate variability (HRV), eye-tracking, electroencephalography (EEG) functional near infrared spectroscopy (fNIRS), and skin conductance [6]. The most used real-time measure of surgeon cognitive load is HRV which objectively measures the balance between the sympathetic and parasympathetic autonomic nervous systems [39]. Heart rate variability (HRV) has been quantified by the analysis of variation of beat-to-beat intervals [15]. The ratio of low-frequency to high-frequency HRV parameters has been used as an objective measure of cognitive load. HRV measurements are different to absolute heart rate parameters which are less sensitive to fluctuations in cognitive load.

Eye-tracking devices measure eye movement and pupil dilation. A review article on the use of pupil metrics to measure cognitive workload broadly categorised the metrics into pupil, blink, and gaze tracking data [11]. These metrics were used as direct measures of cognitive workload, determination of expertise level, and predictors of performance. Pupil metrics provide a reliable Index of cognitive activity. Pupil diameter, task-evoked pupillary response, blink rate, eye tracking, and microsaccades have been used to describe pupil metrics. Studies have used head-mounted, screen-based, and embedded eye trackers as well as EEG and electrooculogram to measure these pupil metrics. Pupil diameter and gaze entropy (index of visual scanning randomness) were positively correlated with workload in one robotic simulation surgery study [40].

EEG has been correlated with surgical performance and NASA-TLX score. Shaiei et al developed an EEG model which combined regional brain network flexibility and a deep convolutional neural network to classify workload levels resulting in an accuracy of over 90% [41]. The model can be used to identify if the surgeon needs more experience in performing motor, cognitive or perception tasks according to calculations of the individual cortices. Multimodal sensing outperformed individual sensors for predicting cognitive load. Lim et al demonstrated that EEG spectral analysis could distinguish the cognitive workload of a surgeon not only relating to surgical task difficulty but also to multi-tasking requirement [42].

Neuroimaging can be used to assess brain function during mentally demanding tasks. The prefrontal cortex is associated with attention, concentration, and task engagement. There is an inverted-U-shaped relationship between prefrontal cortex activation and mental workload, with initial increase and subsequent decrease with excessive levels of CWL [43]. Using fNIRS, Singh et al found robotic suturing was associated with greater prefrontal activation compared with laparoscopic suturing [44]. Skin conductivity response has been found to correlate with physiological arousal state and sympathetic activity [16]. Skin conductance has been correlated with perceived mental stress, blink rate, EEG activity, and intraoperative performance.

Automated performance metrics are used to assess technical skills. Some kinematic measures may be effective for cognitive-motor skill evaluation in robotic surgery [45, 46]. Mental workload may be decreased when the biological two-thirds power law based on the relationship between velocity and radius of curvature of trajectory is obeyed. In addition, the combination of speed and accuracy is a reliable measure of cognitive performance [47]. Analysis of these two parameters need to be considered together because of the speed-accuracy trade-off according to Fitt’s law, which predicted that the time to rapidly move to a target area is a function of the ratio between the distance to the target and the width of the target [48]. This law has also been confirmed in robotic simulation studies [49].

## Cognitive training

Cognitive training can play a crucial role in the process of skill acquisition during the three Fitts and Posner stages of motor learning: from cognitive to integrative and then to autonomous [45]. Considerable cognitive resources are required in the initial stages when the surgeon is learning how to complete tasks. The integrative stage involves more attention on performance than strategy. Finally, cognitive load is low when tasks can be performed automatically with mastery of motor skills.

Cognitive training can improve skills such as attention, working memory, problem solving, attentional stability and self-confidence [50]. It may have long-term effects on improving the speed and logic of processing information. Constructed schemas can become automated if they are repeatedly applied, with the automation freeing up working memory. Experts develop schemas to incorporate the interacting elements to reduce intrinsic CWL. Decreasing extraneous load (influenced by how tasks are presented), managing intrinsic load (relating to the number of elements to be processed simultaneously in working memory), and optimising germane load (relating to the act of learning) can reduce CWL [5]. With robotic surgery training, starting with medium fidelity simulations on the computer simulator and progressing to the high fidelity real environment of operating on patients can be used to manage the intrinsic load by gradually increasing the number of interactive elements [5].

Mentorship, technical practice and watching videos are the most common traditional cognitive training methods in surgery [45]. Mentorship by an expert can be performed readily using the dual console robotic system, with real-time assessment and constructive feedback [51]. Mentorship does not only involve motor learning for technical skills but also development of cognitive skills. Studies have shown that simulation training has good content, face, construct, and concurrent validity. It is a useful realistic training tool which can distinguish expertise levels and correlates closely with expert assessments. Additional mentoring during simulation training has been demonstrated to reduce mental workload [52]. This may reduce extraneous load by avoiding trial and error through proper guidance. Novice and expert robotic surgeons have been shown to use video review to increase efficiency, refine technical skills, and for reflective practice [53]. Kim et al. reported that trainees preferred video-based curriculum over books for robotic surgery preparation and 19 of the 20 subjects felt that the videos helped them with the cognitive aspects of the surgery [54].

Cognitive training can also occur with computer-based cognitive simulation, mental rehearsal, or cognitive task analysis. Julian et al developed a computer-based intelligent tutoring system to train the cognitive skills of novice surgeons to complete basis robotic suturing [55]. It involved an adaptive course (which was dependent on the learner’s knowledge and engagement) and multimodal task analysis. Mental training via the use of motor imagery has been used in cognitive training and involves mental rehearsal of a motor task without physical execution. In one study, motor imagery was found to be an effective training tool for improving technical but not non-technical skills in robotic surgery [56]. The PETTLEP model was used to develop an effective motor imagery script incorporating the seven domains of Physical, Environment, Task, Timing, Learning, Emotion, and Perspective. There was a 1.7 mean benefit in Global Evaluative Assessment of Robotic Skills (GEARS) score with cognitive training. GEARS has been shown to be a valid and reliable measure of robotic technical skills [57, 58]. GEARS is composed of six domains: depth perception, bimanual dexterity, efficiency, force sensitivity, autonomy and robotic control. The improvement in GEARS scores involved tasks more associated with the cognitive challenges of robotic surgery rather than purely technical skills. The authors postulated that the absence of improvement in non-technical skills was due to the more abstract nature of non-technical skills which prevented effective mental visualisation and training.

Cognitive task analysis (CTA) involves expert analysis of the cognitive processes behind each key step of an operation [59]. CTA is dependent on expert opinion with regards to task sequence, key decision points and rationale, technical tips, potential pitfalls, and strategies to avoid and manage problems. However, experts often do not verbalise their cognitive processes well [53]. A meta-analysis found that CTA-based training was significantly more effective than conventional training in improving technical performance [59]. The authors hypothesised that CTA-training provided learners a structured framework to supplement the cognitive stage of motor learning. Nine cognitive behaviours were revealed (respect for patient-specific factors, tactical modification, adherence to core surgical principles, task completion, judicious technique and instrument selection, visuospatial awareness, team-based communication, anticipation and forward planning, finessed tissue handling) in a CTA of a robotic procedure [60].

## Assessment of cognitive skills

Cognitive assessment may offer a more effective way to differentiate robotic expertise level than automated performance (tool-based) metrics. Guru et al found that expert robotic surgeons displayed lower high-level cognitive engagement and lower cognitive workload as measured by EEG [61]. High-level engagement may be more important during the initial stages of learning and reflects attentive focus. Lower cognitive workload in experts may free up neural networks to help with processing of new information, speed-up decision-making, and improve task execution. The learned behaviour of surgical proficiency developed over time with practice may diminish the role of cognition. In another study, Guru et al reported that expert surgeons utilize different mental resources according to their need [8]. More mental resources were used in complex unfamiliar situations whereas working memory overload were avoided during uncomplicated surgical tasks with the use of pattern recognition and muscle memory.

## Conclusion

The relationship between cognition and robotic surgery is complicated and multi-faceted. The effect of robotic surgery on CWL is complex because the postural, visualisation, and manipulation ergonomic benefits for the surgeon may be offset by the disadvantages for the team with separation and reduced surgeon situation awareness. Physical fatigue and workflow disruptions have a negative impact on CWL. Physiological measurements of CWL are more precise and time-sensitive compared with subjective rating scores. Cognitive training has the potential to hasten the progress of motor learning. Finally, assessment of cognitive skills may be more reliable than tool-based metrics in the assessment of the learning curve.

## Data Availability

No datasets were generated or analysed during the current study.
